# Assortment, but not knowledge of assortment, affects cooperation and individual success in human groups

**DOI:** 10.1371/journal.pone.0185859

**Published:** 2017-10-02

**Authors:** Jaakko Junikka, Lucas Molleman, Pieter van den Berg, Franz J. Weissing, Mikael Puurtinen

**Affiliations:** 1 Department of Biological and Environmental Science, University of Jyväskylä, Jyväskylä, Finland; 2 Centre of Excellence in Biological Interactions, University of Jyväskylä, Jyväskylä, Finland; 3 Centre for Decision Research and Experimental Economics, School of Economics, University of Nottingham, University Park, Nottingham, United Kingdom; 4 Center for Adaptive Rationality, Max Planck Institute for Human Development, Berlin, Germany; 5 Groningen Institute for Evolutionary Life Sciences, University of Groningen, Groningen, Netherlands; 6 Lab of Socioecology and Social Evolution, KU Leuven, Leuven, Belgium; 7 Netherlands Institute for Advanced Study in the Humanities and Social Science (NIAS-KNAW), Amsterdam, Netherlands; Middlesex University, UNITED KINGDOM

## Abstract

The success or failure of human collective action often depends on the cooperation tendencies of individuals in groups, and on the information that individuals have about each other’s cooperativeness. However, it is unclear whether these two factors have an interactive effect on cooperation dynamics. Using a decision-making experiment, we confirm that groups comprising individuals with higher cooperation tendencies cooperate at a higher level than groups comprising individuals with low cooperation tendencies. Moreover, assorting individuals with similar cooperation tendency together affected behaviour so that the most cooperative individuals tended to cooperate more and the least cooperative individuals cooperated less, compared to their behaviour in randomly formed groups. In line with predictions of evolutionary models of cooperation, there was a strong positive association between individuals’ cooperation tendency and success when groups were formed assortatively, whereas such association did not exist when groups were formed randomly. Surprisingly, information about group members’ cooperativeness in assorted groups had no effect on cooperation levels. We discuss potential explanations for why information about cooperativeness of others may be disregarded in certain circumstances.

## Introduction

The evolution of cooperation has been challenging to explain because free-riding individuals, who reap the benefits of cooperation without contributing to its costs, will often achieve higher payoffs than cooperators [1,2]. Explanations of human cooperation have largely been based on reciprocity, in which cooperation is directed to those who are expected to cooperate in kind. Reciprocity relies on information about the previous behaviour of interaction partners [3–6]. An individual can gather information about the cooperativeness of others through personal interaction [7,8], or through third parties [9,10]. Many experiments have shown that humans mainly cooperate with those who have cooperated with them in the past [11,12], or with those who have been cooperative in interactions with third parties [12–15].

Gathering information on others’ cooperativeness is critical for social decision making: studies from a range of disciplines have shown that there are individual differences in human cooperation tendencies [12,16–22], and these tendencies are consistent across time and context [19,21,23–25]. Evolutionary theories predict that this variation in human cooperativeness plays an important role in the performance of groups [26,27]. Empirical evidence, by and large, confirms these predictions: when individuals with similar cooperation tendencies group together through positive assortment, cooperators may thrive as they can enjoy the benefits of cooperation without being exploited by less cooperative individuals [17,21,28,29].

Despite the importance of assortment and information about partner cooperativeness for the evolution of human cooperation, it remains poorly understood how these two factors interact. De Oliveira et al. [30] found that in assorted groups, cooperative decisions of both the most and the least cooperative individuals were insensitive to information about the cooperation tendencies of their group members. In addition, work by Gächter and Thöni [31,32] suggests that information on the similarity of group members’ past cooperative decisions has no effect on overall cooperation level. As these findings contrast with numerous studies showing the substantial effect of information on others past cooperativeness (e.g. 11–14), additional experiments are needed to unravel the interplay of information and assortment.

Here we study how assorting individuals with similar cooperation tendencies to groups, and information about group members’ cooperativeness in assorted groups, affect cooperation and the success of individuals in a public goods game. Specifically, we conducted a decision-making experiment to test three main hypotheses: i) assortment leads to differences in the level of cooperation across groups comprising different types of individuals; ii) assortment leads to a positive association between individual cooperation tendency and earnings in the game, whereas such an association should not exist in the absence of assortment; iii) providing information on the nature of assortment amplifies the differences in cooperation levels between assorted groups.

These hypotheses are based on previous findings that individual differences in cooperativeness are consistent [19,23] and that individuals tend to adjust their cooperation to the anticipated cooperation of their group members as suggested by models of direct and indirect reciprocity (e.g. 5,14,16). Thus, as cooperation depends on the anticipated cooperation of others, informing the most cooperative individuals that they will interact only with other highly cooperative individuals should fuel trust and cooperation through anticipated reciprocity. Similarly, informing the least cooperative individuals that they will interact with other non-cooperators should create anticipation of defection, leading to lower levels of cooperation.

We show that assorting individuals into groups based on individual cooperativeness had substantial effects on the level of cooperation within groups, partly because assortment seemed to make more cooperative individuals cooperate more and the least cooperative individuals cooperate less, compared to a random grouping setting. Further, we found a consistent positive association between individuals’ cooperation tendency and success when groups were formed assortatively, but not when groups were formed randomly. Surprisingly, however, we found that individuals were insensitive to the provided information on the composition of their group.

## Methods

To study the effects of assortment and information about assortment on cooperation in groups, we conducted an experiment based on the public goods game (PGG). The PGG reflects a situation in which temptation to free-ride makes it difficult to maintain individually costly cooperation. In a linear PGG, the average payoff per group member is maximized if all group members cooperate, that is if everybody contributes maximally to the public good. Yet, any personal contribution decreases the net payoff to the individual group member. Therefore, in each single round of the PGG, individual payoff is maximised by contributing nothing and profiting from the contributions of others.

In each of our experimental sessions, sixteen subjects were arranged in groups of four, in which they repeatedly played a PGG. At the beginning of each round, each subject was allocated 20 points to distribute between a group project and their personal account. After all four subjects had made their decisions, the total contributions to the group project were doubled and divided equally among the group members, irrespective of their contributions. After each round, subjects were presented with the anonymized contributions and earnings of each of their fellow group members.

The experimental setup consisted of two stages. In Stage 1, subjects interacted over ten rounds of a PGG, with group composition randomly changing after every round. This means that subjects essentially played a series of one-shot PGGs. In this setup, there are no possibilities for strategic cooperation or build-up of reputation, and contributing nothing is the dominant strategy. Accordingly, we interpret the average contribution level (on the range 0–20) over all rounds of Stage 1 as a measure of a subject’s cooperation tendency. Subjects in each session were classified to four ‘cooperative tiers’ based on their behaviour in Stage 1, with four subjects with the highest cooperation tendency belonging to tier 1, the four second most cooperative to tier 2, the four third most to tier 3, and the four least cooperative to tier 4. The subjects did not know that Stage 1 was designed to measure their cooperation tendency, nor were they aware of the nature of Stage 2 before it began. In the beginning of the experiment, the subjects were only informed that the study would continue after Stage 1 and that they would receive new instructions on their computer screen after completing Stage 1.

**Fig 1:**
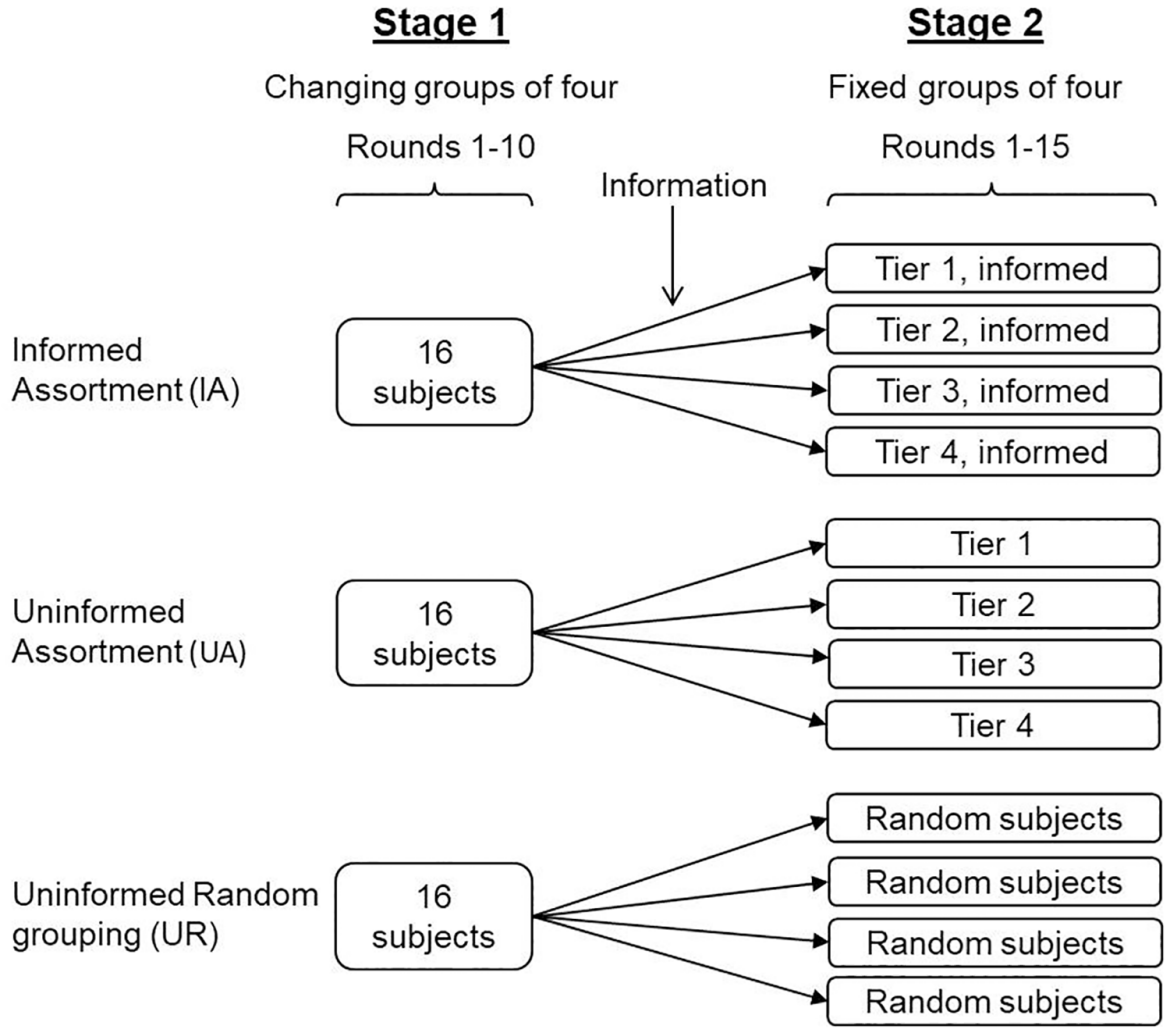
Experimental procedure. Subjects played a total of 25 rounds of the PGG in three experimental treatments: Informed Assortment (IA), Uninformed Assortment (UA) and Uninformed Random grouping (UR). Each treatment was replicated 5 times. Each of the 15 replicates was run in a session involving 16 subjects. In Stage 1, the subjects played 10 rounds of the PGG in groups of four where group composition changed randomly after each round. After Stage 1, all subjects were informed that the groups were fixed for the next 15 rounds of PGG of Stage 2. In the IA and UA treatments the four fixed groups were formed by assorting the 16 subjects according to their mean contributions in the 10 rounds of Stage 1. Only in the IA treatment, subjects received the additional information that the groups were assorted according to cooperativeness, and to which cooperative tier they were allocated. In the UR treatment, the four fixed groups were formed at random.

Stage 2 was designed to study if assorting individuals with similar cooperation tendencies affects cooperation and if information about previous cooperativeness of group members affects cooperation in assorted groups. In Stage 2, subjects interacted additional 15 rounds of the PGG, this time with group composition remaining fixed over all rounds. We implemented three treatments (Fig 1): Informed Assortment (IA), Uninformed Assortment (UA) and Uninformed Random grouping (UR). To test if assortment affects cooperation of individuals from different cooperative tiers we compared treatments UA and UR, and to test if information about assortment affects cooperation in assorted groups we compared treatments IA and UA.

In treatments IA and UA, subjects were assorted into groups based on their cooperativeness in Stage 1 so that individuals from the same cooperative tier in Stage 1 were grouped together. In treatment IA, subjects were informed at the beginning of Stage 2 about the assortment procedure and to which cooperative tier they belonged (e.g. “Now all players are ranked and grouped according to their contributions in the first ten rounds. The player that contributed most is ranked #1 and the player contributed the least is ranked #16. You are in the group of players ranked 13–16”). In treatment UA, the subjects were informed that the groups were now fixed, but they were not informed about the assortment procedure. In treatment UR, groups were formed randomly with respect to the cooperative tier in Stage 1, and the subjects were only informed that the groups were now fixed.

The experiment consisted of fifteen sessions, with five sessions for each of the three treatments (sixteen subjects in each session, total N = 240; 71% females (50–94% per session), aged 19–33 years, mostly university students across variety of disciplines, 68% had earlier experience with economic experiments. The level of cooperation in the first round did not differ between male and female subjects (ANOVA: F_1,190_ = 0.123, p = 0.726) and was not impacted by earlier experience with participation in economic experiments (F_1,190_ = 2.367, p = 0.126). Subjects gave written informed consent before participating. The experimental setup was approved by the Sociological Laboratory of the University of Groningen. We followed the guidelines established by the VSNU Code of Conduct for Scientific Practice when running experimental sessions.

Instructions for the experiment were read out loud by the experimenter at the start of each session. Instructions for the two separate stages, including the number of rounds, were explained to subjects on their computer screens at the beginning of each stage. Before the start of each stage, subjects filled out a short quiz to check their comprehension. Sessions lasted about one hour and subjects earned on average €14.87 (ranging from €10.56 to €18.35). Subjects were paid in cash at the end of the experiment and were unaware of the earnings of others. The experiment was programmed and conducted with the experiment software z-Tree [33] (code available upon request from the first author). All statistical analyses were conducted with SPSS (v. 20.0.0.2) and R (v. 3.1.3).

## Results

**Fig 2:**
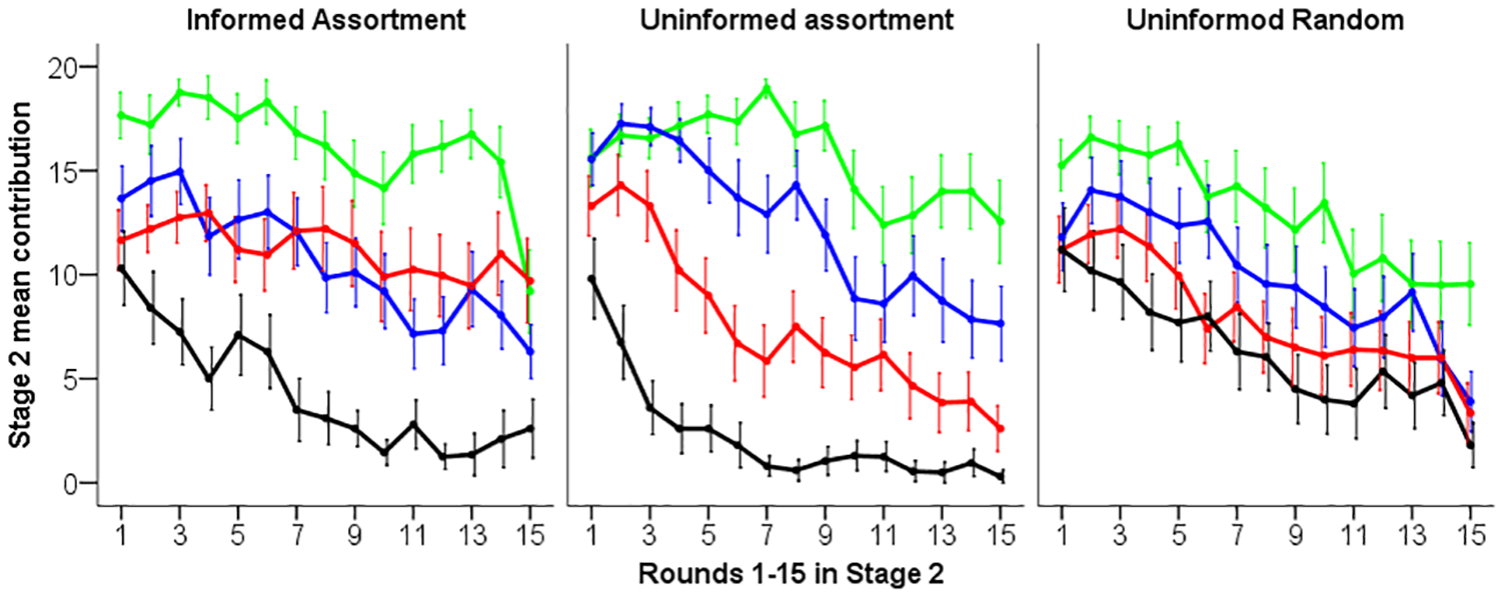
Cooperation by subjects from different cooperative tiers in assorted and random groups in Stage 2. In treatments Informed Assortment and Uninformed Assortment, groups were formed by assorting subjects according to their cooperation tendencies measured in Stage 1. In the Uninformed Random grouping treatment, groups were formed randomly. Note that while in treatments Informed Assortment and Uninformed Assortment subjects in a group belong to the same cooperative tier, in the Uninformed Random treatment groups comprised individuals from different cooperative tiers. Lines indicate mean contribution of individuals belonging to the same cooperative tier across five replicate sessions (green: tier 1, blue: tier 2, red: tier 3, black: tier 4). Error bars indicate ±1 s.e of individual contributions. See Table 1 for statistical analysis of effects of information and assortment on contribution levels.

As expected, when individuals with similar cooperation tendencies were grouped together, there were strong differences in the level of cooperation between groups, depending on the cooperative tier of the subjects (Fig 2 treatments IA and UA, Table 1). In Stage 2, cooperation levels in assorted groups corresponded to the ‘cooperative tiers’ assigned to individuals in Stage 1 (with tier 1 having the highest cooperation level, followed by 2, 3 and then 4).

**Table 1:** Anova based on Linear Mixed Models (LMMs) fitted to contributions in Stage 2, using ‘subject nested in group’ as a random effect. The model estimates are given in the Supporting Information (S1 Table). The analysis includes a comparison of (i) Uninformed Assortment and Uninformed Random grouping to study the effect of assortment and (ii) a comparison of Informed Assortment and Uninformed Assortment to study the effect of information. The factors “Assortment” and “Information” compare treatments. ‘Round’ is the round number (1–15) to account for time trends in cooperation levels in Stage 2 (Fig 2) and ‘Cooperative tier’ is a factor reflecting individuals’ tier rank (1, 2, 3 or 4) based on cooperation in Stage 1. The significant interaction between ‘Assortment’ and ‘Cooperative tier’ results from larger differences between individuals with the highest and lowest cooperation tendencies in assortment compared to the random grouping treatment (i.e., assortment amplifies existing cooperation tendencies). Surprisingly, the effect of ‘Information’ is not significant. In each treatment, there were 80 subjects (five sessions with 16 subjects in each session).

**Uninformed Assortment vs** **Uninformed Random grouping**	**SS**	**MS**	**df**	**F**	**p**
Assortment	0.10	0.10	1	0.00	0.96
Round	14876.50	14876.50	1	572.74	< 0.01
Cooperative tier	755.80	251.90	3	9.70	< 0.01
Assortment * Cooperative tier	261.40	87.10	3	3.36	0.03
**Informed Assortment vs** **Uninformed Random grouping**	**SS**	**MS**	**df**	**F**	**p**
Information	20.50	20.50	1	0.68	0.42
Round	11400.90	11400.90	1	377.34	< 0.01
Cooperative tier	1283.60	427.90	3	14.16	< 0.01
Information * Cooperative tier	60.80	20.30	3	0.67	0.58

**Fig 3:**
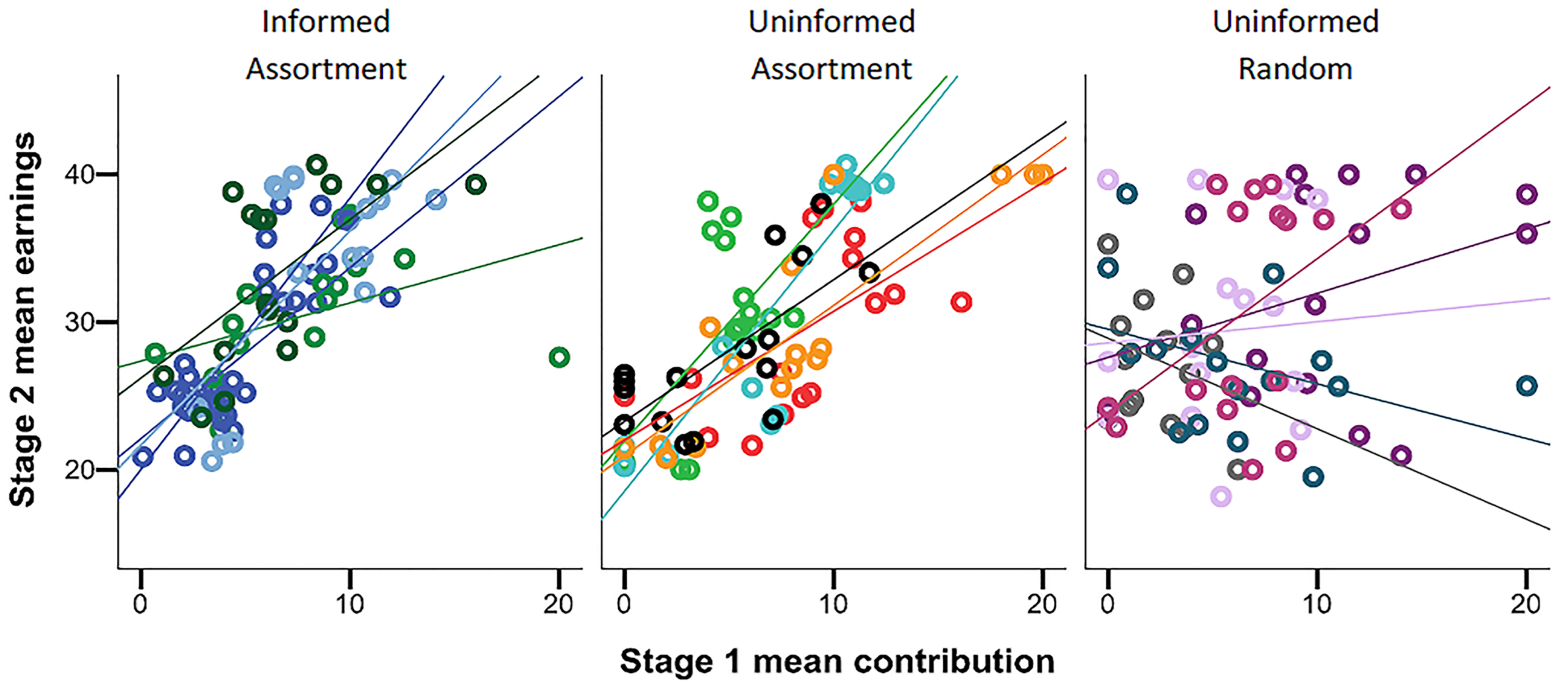
Association between cooperativeness and earnings. The relationship between individual mean contribution in Stage 1 (measuring cooperation tendency) and mean earnings in Stage 2 (measuring success) for each of the three treatments. Coloured dots indicate individuals from each session (each session comprising 16 individuals) and lines indicate linear regressions for each session.

As a consequence of the between-tier differences in cooperation levels in assorted groups (treatments UA and IA), there was a positive association between individual cooperation tendency measured in Stage 1 and earnings in Stage 2 (Fig 3). However, such an association was not observed when groups were formed randomly (treatment UR). The association between cooperation tendency and earnings was quantified by linear regression coefficient of earnings in Stage 2 on mean contributions in Stage 1 for each experimental session (*β*_Treatment_ = mean regression coefficient, lower and upper 95% confidence limits in parentheses: *β*_IA_ = 1.78 (0.78, 2.78); *β*_UA_ = 1.88 (1.22, 2.64); *β*_UR_ = 0.19 (-1.03, 1.41)). The regression coefficients differed significantly between the treatments (ANOVA: F_2, 14_ = 6.70, p = 0.01), and a Tukey test revealed that the coefficients in the random grouping treatment differed significantly from those in both assortment treatments (UR vs IA: p = 0.024, UR vs UA: p = 0.018).

**Fig 4:**
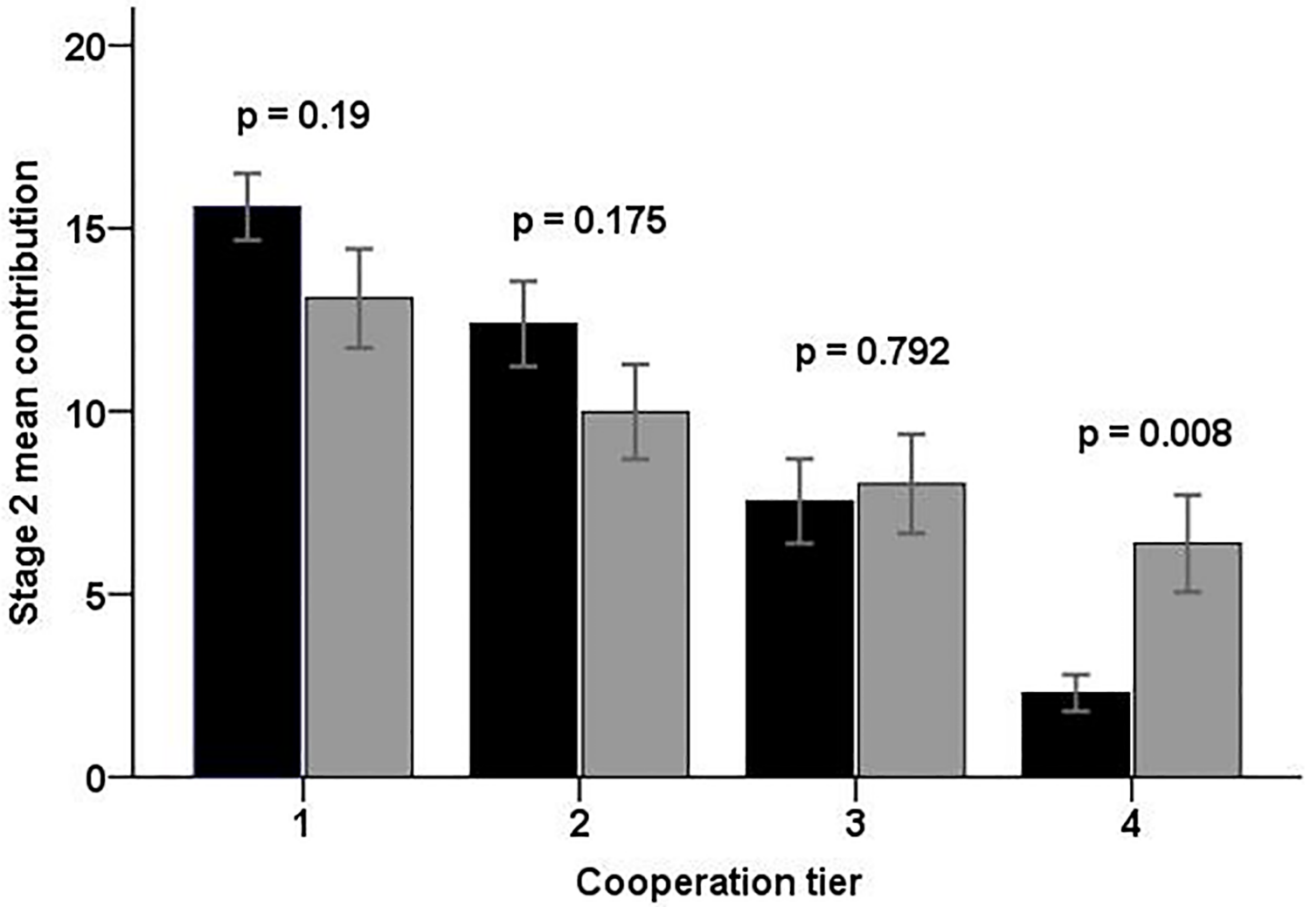
Effect of assortment on cooperation for the four cooperative tiers. Black bars represent the individuals in the Uninformed Assortment treatment and grey bars represent the individuals in the Uninformed Random treatment. Significance values refer to independent sample t-tests (not assuming equal variances) comparing contributions within each cooperative tier. Error bars indicate ±1 s.e.

Moreover, assortment modulated the cooperative behaviour of individuals. As can be seen in Figs 2 and 4, the differences in the level of cooperation of individuals belonging to different cooperative tiers were larger when groups were formed by assortment than when they were formed at random. This result is corroborated by a statistically significant interaction between the effects of assortment and cooperative tier on mean contributions in Stage 2 (Table 1; p = 0.03). The strongest effect of assortment on cooperation was observed among the least cooperative individuals (tier 4; see Fig 4).

However, contrary to our expectations, informing individuals about the assortment procedure and the cooperativeness of fellow group members did not affect overall or per-tier levels of cooperation in assorted groups (Table 1: comparison between treatments UA and IA; ‘Information’ and interaction between ‘Information’ and ‘Cooperative tier’ are not significantly different from zero). In all tiers, cooperation decreased over time (Fig 2).

In order to explicitly test if information on the assortment procedure affected cooperation immediately after this information was received, we compared mean contributions in the first round of Stage 2 in each cooperative tier in treatment UA with those in treatment IA (Table 2). However, even in this restricted case, information on cooperativeness had no effect on the cooperation of any tier.

**Table 2:** Comparison of individual contributions in the first round of Stage 2 between Informed Assortment (IA) and Uninformed Assortment (UA). For each cooperative tier, we show the mean and standard error of individual contributions pooled over the five replicates. The group identity is not included in the analysis because the decisions of subjects in a group can be considered independent in the first round of Stage 2. The average contribution does not differ significantly between treatments in any of the four tiers (independent-samples t-test).

	Informed Assortment (IA)		Uninformed Assortment (UA)			
Cooperative tier	mean	s.e.	mean	s.e.	t_38_	p
1st	17.65	1.10	15.60	1.38	1.16	0.25
2nd	13.65	1.57	15.55	1.26	-0.94	0.35
3rd	11.65	1.44	13.30	1.43	-0.81	0.42
4th	10.30	1.77	9.80	1.92	0.19	0.85

## Discussion

In line with earlier studies [28,29,34], we found that when individuals are assorted to groups based on their cooperativeness, groups comprising more cooperative individuals are able to maintain higher levels of cooperation than groups comprising less cooperative individuals. However, as the level of cooperation slightly declined even in the most cooperative assorted groups, experiments with even more interaction rounds are needed to determine if differences in cooperation are maintained in the long run. We also demonstrate that in assorted groups, differences in the level of cooperation resulted in a consistent positive association between individual cooperation tendency and success. In contrast, when groups were formed randomly, there was no consistent association between individual cooperation tendency and success, as in the study by Kurzban and Houser [19]. These results support evolutionary theories holding that positive assortment is important for the success of cooperation [26,27].

Interestingly, we find that assortment influenced cooperation so that the individuals with the highest cooperation tendencies were inclined to cooperate even more when assorted with other cooperators, and the individuals with the lowest cooperation tendencies cooperated at an even lower level when grouped with other non-cooperators, in comparison to the situation where groups were formed randomly with respect to subjects’ cooperation tendencies. This finding is in line with results of Van den Berg et al. [22], who found that individuals with lowest cooperation tendencies are likely to follow the example of the least cooperative behaviour in a group, whereas the most cooperative individuals tended to follow the example of more cooperative behaviour. These different responses to the behaviour of others may partly explain the strong differences in levels of cooperation among different cooperative tiers in the assorted groups.

Contrary to our expectations, information about assortment and cooperativeness of fellow group members did not affect the level of cooperation or the success of individuals with different cooperation tendencies. The provided information did not influence individual cooperative decisions of individuals from any cooperative tier even in the first round of Stage 2, immediately after the information was received (Table 2). Our result is in line with Gächter and Thöni (2011), who found no effect of knowledge of being grouped with “like-minded” cooperators on mean cooperation. Our result is also in line with De Oliveira et al. (30), who found that providing information about the heterogeneity of cooperative types (conditional cooperators and selfish types) within groups did not affect cooperation in either homogenous or heterogeneous groups. Our study differed from De Oliveira et al.’s (30) in three main respects: i) the information given (previous cooperativeness and assortment vs. variation in cooperative type), ii) in composition of groups (whole population with homogenous groups of cooperative tiers vs. population subset of selfish and conditional cooperator types in heterogeneous and homogeneous groups) and in iii) group size (4 instead of 3). Even so, both studies arrive at similar conclusions, suggesting that our findings do not reflect specifics of the experimental implementation.

Our finding that information concerning the previous behaviour of group members has no effect on cooperation seems surprising in view of numerous other studies stressing the importance of such information for cooperative decision making (e.g. 6,8,9,11). One possible reason why information about past cooperation did not have an effect in our study may be connected to the timing of information provision. As in the study of de Oliveira et al. [30], also we provided information about group composition at the beginning of a new interaction stage. At the beginning of a new interaction stage, people tend to return to their original levels of cooperation, even after experiencing the collapse of cooperation in previous interactions, a phenomenon known as ‘restart effect’ [35,36]. These previous findings, together with our results, suggest that people neglect information about the cooperativeness of others from previous stages, or at least they are not sensitive to this information when a new stage begins.

Attempts to signal one’s cooperativeness at the start of new series of interactions may give rise to such restart effects [37]. Evolutionary models based on the handicap principle [38] suggest that such costly signaling can function as the basis for partner choice, enabling cooperators to assort and form new successful cooperative interactions [37,39–41]. Less cooperative individuals could also benefit from such signaling, as it is in their interest to be part of a cooperative group. Another mechanism that may have contributed similar cooperation levels in informed and uninformed assorted groups is the so-called False Consensus Effect [42,43], which states that people tend to deem their own behaviour common and appropriate, and accordingly believe that others will behave in a similar way. If this were the case in our experiment, then subjects in our Uninformed Assortment treatment already believed that others were similar to themselves and information provided in our Informed Assortment treatment would only have confirmed pre-existing beliefs.

To conclude, our experiment showed that assorting individuals into groups based on varying individual cooperation tendencies have a substantial effect on groups’ ability to achieve cooperation. This was partially caused by assortment amplifying individual differences in cooperation, particularly due to the collapse of cooperation in groups comprising only individuals with lowest cooperation tendencies. Further, assortment had significant effects on the earnings of individuals: cooperators outperformed non-cooperators in the presence of assortment, but not when grouping was random. Surprisingly, however, individuals by all cooperation tiers were insensitive to the provided information on group composition, suggesting that humans are facultative users of potentially critical information.

## Supporting information

S1 Table Determinants of cooperation in fixed groups. Both models present estimates of a Linear Mixed Model fit to individual contributions in stage 2, with ‘subject nested in group’ as a random effect. Model 1 focuses on the effects of assortment by comparing the Uniformed Assortment and the Uniformed Random treatments. ‘Uninformed Random’ and ‘tier 4’ are the baseline categories. Model 2 focuses on the effects of information about assortment by comparing the Informed Assortment and Uninformed Assortment treatments. ‘Uninformed Assortment’ and ‘tier 4’ are the baseline categories. Significance codes: * p < 0.05, ** p < 0.01, *** p < 0.001.
(DOCX)

S1 Raw Data (TXT)
