# Supplementary material for: Assortment, but not knowledge of assortment, affects cooperation and individual success in human groups
Source: PLoS One. 2017 Oct 2;12(10):e0185859. doi: 10.1371/journal.pone.0185859 (PMC5624630; doi:10.1371/journal.pone.0185859)
Supplement: S1 Table — Both models present estimates of a Linear Mixed Model fit to individual contributions in stage 2, with ‘subject nested in group’ as a random effect. Model 1 focuses on the effects of assortment by comparing the Uniformed Assortment and the Uniformed Random treatments. ‘Uninformed Random’ and ‘tier 4’ are the baseline categories. Model 2 focuses on the effects of information about assortment by comparing the Informed Assortment and Uninformed Assortment treatments. ‘Uninformed Assortment’ and ‘tier 4’ are the baseline categories. Significance codes: * p < 0.05, ** p < 0.01, *** p < 0.001. (DOCX) [file pone.0185859.s001.docx]

|  | Model 1 | Model 2 |
| --- | --- | --- |
| Round | -0.58 (0.02)*** | -0.50 (0.03)*** |
| Tier 1 | 4,27 (0.72)*** | 13.29 (2.78)*** |
| Tier 2 | 1,54 (0.69)* | 10.09 (2.78)*** |
| Tier 3 | 1,99 (0.71)** | 5.24 (2.78) |
| Assortment | -5.12 (2.63) |  |
| Tier 1 * Assortment | 9.02 (3.36)* |  |
| Tier 2 * Assortment | 8.55 (3.36)* |  |
| Tier 3 * Assortment | 3.25 (3.36) |  |
| Information |  | 2.04 (2.78) |
| Tier 1 * Information |  | -1.42 (3.93) |
| Tier 2 * Information |  | -3.77 (3.93) |
| Tier 3 * Information |  | 1.60 (3.93) |
| (Intercept) | 6.33 (1.98)** | 12.03 (1.25)*** |
